# Comparative Analysis of the Therapeutic Effects of MSCs From Umbilical Cord, Bone Marrow, and Adipose Tissue and Investigating the Impact of Oxidized RNA on Radiation-Induced Lung Injury

**DOI:** 10.1155/2024/7419270

**Published:** 2024-10-24

**Authors:** Rui Zhai, Fumin Tai, Kexin Ding, Xin Tan, Hujie Li, Zhengyue Cao, Changhui Ge, Xiaofei Zheng, Hanjiang Fu

**Affiliations:** ^1^School of Basic Medical Sciences, Anhui Medical University, Hefei 230032, China; ^2^Department of Experimental Hematology and Biochemistry, Beijing Key Laboratory for Radiobiology, Beijing Institute of Radiation Medicine, Beijing 100850, China; ^3^Department of Radiation Oncology, Chinese PLA General Hospital, Beijing 100853, China

## Abstract

Radiation-induced lung injury (RILI) is frequently observed in patients undergoing radiotherapy for thoracic malignancies, constituting a significant complication that hampers the effectiveness and utilization of tumor treatments. Ionizing radiation exerts both direct and indirect detrimental effects on cellular macromolecules, including DNA, RNA and proteins, but the impact of oxidized RNA in RILI remains inadequately explored. Mesenchymal stem cells (MSCs) can repair injured tissues, and the reparative potential and molecular mechanism of MSCs in treating RILI remains incompletely understood. This study aimed to investigate the therapeutic effects and mechanisms of action of three distinct sources of MSCs, including human umbilical cord mesenchymal stem cells (UCMSCs), bone marrow mesenchymal stem cells (BMSCs), and adipose-derived stem cells (ADSCs), in thoracically irradiated mice. Comparative analysis revealed that all three types of MSCs exhibited the ability to mitigate radiation-induced inflammatory infiltration, alveolar hemorrhage, and alveolar wall thickening in the lung tissue of the mice. MSCs also attenuated RILI by decreasing inflammatory factors, upregulating anti-inflammatory factor expression, and reducing collagen accumulation. Immunohistochemical results showed that all three MSCs reduced radiation-induced cell apoptosis and promoted the regeneration of lung tissue cells. The analysis of malondialdehyde (MDA) and 8-hydroyguanosine (8-OHG) content indicated that MSCs possess reparative properties against radiation-induced oxidative damage in lung tissue. The study provides evidence that UCMSCs are a more appropriate therapeutic option for RILI compared to BMSCs and ADSCs. Additionally, MSCs effectively reduce the accumulation of oxidized RNA in RILI, thereby, presenting a unique avenue for investigating the underlying mechanism of MSC-based treatment for RILI.

## 1. Introduction

Radiation-induced lung injury (RILI) is a common adverse reaction after radiotherapy, which limits the therapeutic dose that can be administered for tumor irradiation [1–3]. RILI is most prevalent among patients treated with radiotherapy in the thoracic radiotherapy department, including patients with lung cancer (5%–25%), followed by mediastinal lymphomas (5%–10%) and breast cancer (1%–5%) [4]. RILI is a complex pathologic process that manifests as early radiation pneumonitis and late radiation pulmonary fibrosis [5]. RILI is a chronic, progressive lung disease, and the precise molecular mechanisms of the development of RILI have not been fully elucidated, while the available medications for RILI are poorly tolerated and have side effects [6]. Therefore, the prevention and treatment of RILI is a priority in radiation medicine.

Mesenchymal stem cells (MSCs) are pluripotent stem cells characterized by multidirectional differentiation, self-renewal, and low immunogenicity [7, 8]. MSCs possess the ability to rescue or repair impaired cells and tissues through diverse mechanisms, and show high potential for clinical application [9]. MSCs have a homing effect, and intravenous injection of exogenous MSCs will primarily home to lung tissue, which is conducive to lung tissue repair [10]. Currently, umbilical cord mesenchymal stem cells (UCMSCs), bone marrow mesenchymal stem cells (BMSCs), and adipose-derived stem cells (ADSCs) are the most used MSC types in clinical trials [11]. The therapeutic effectiveness of MSCs in RILI has been substantiated [12–14]. However, there are fewer reports on the differences in the therapeutic outcomes of MSCs derived from human different sources in the context of RILI.

Radiation exposure leads to the generation of substantial quantities of reactive oxygen species (ROS) in tissues, resulting in DNA damage [1, 6]. However, the investigation of radiation-induced RNA damage has received comparatively less attention. Hydroxyl radicals possess the ability to oxidize RNA, and more than 20 different types of hydroxyl radical damage have been identified, while the most prevalent oxidizing base in RNA is 8-hydroxyguanosine (8-OHG) [15, 16]. It has been reported that the level of RNA oxidation in human skin fibroblasts increased significantly following exposure to ultraviolet A radiation, but the level of DNA oxidation did not [17]. However, the level of oxidized RNA in RILI has not been reported. MSCs attenuate oxidative stress induced by lung tissue injury by increasing both superoxide dismutase (SOD) and glutathione (GSH) [18, 19], but the complex and diverse molecular mechanisms of MSCs treatment of RILI are not fully understood.

This study focused on the therapeutic effect of human MSCs from different sources on lung tissue injury in mice after radiation and analyzed the level of oxidized RNA in the lung tissue with RILI and after MSCs treatment, which provides the basis for the use of MSCs in the treatment of RILI.

## 2. Materials and Methods

### 2.1. Animals

Forty male C57BL/6 mice aged 6–8 weeks were obtained from Vital River Experimental Animal Company (Beijing, China). They were randomly assigned to the following five groups (*n* = 8):(A) normal group (NC); (B) irradiation group (IR); (C) IR injected with UCMSC (IR + UCMSC); (D) IR injected with BMSC (IR + BMSC); and (E) IR injected with ADSC (IR + ADSC), which were kept in the experimental animal center. After adequate acclimatization, the mice in groups B–E were subjected to a single dose of 20 Gy (74.62 cGy/min) irradiation using ^60^Co at the Beijing Institute of Radiation Medicine, targeting the lung. Subsequently, groups C–E received an injection of 1 × 10^6^ cells of the corresponding MSCs into their tail veins, within 3 h after irradiation. MSCs were resuspended using phosphate-buffered saline (PBS) buffer. Group B was injected with PBS buffer as control treatment. The mice were weighed throughout the study period. Four mice were sacrificed at 4 and 12 weeks postradiation to obtain lung tissue for analysis. All animal experiments were conducted according to protocols approved by the Institutional Animal Care and Use Committee (IACUC) at Animal Center in Academy of Military Medical Science (Beijing, China).

### 2.2. Cell Culture

The human UCMSCs (DF-GMP-ZB09BA) were cultured with special medium for human UCMSCs (ZQ-1320); human BMSCs (ZQ-0308) and ADSCs (ZQ-0309) were cultured with special medium for MSCs (ZQ-1318). All MSCs were obtained from Zhongqiao Xinzhou Co., Ltd., and incubated at 37°C in a humidified atmosphere with 5% CO_2_.

### 2.3. Lung Histology and Immunohistochemistry

The left lungs of mice were fixed in 4% paraformaldehyde (G1101, Servicebio, China) for 48 h and then embedded in paraffin. Slices with a thickness of 5 μm were prepared and stained with hematoxylin and eosin (H&E), Masson, and immunohistochemistry with terminal deoxynucleotidyl transferase-mediated dUTP nick end labeling (TUNEL) and Ki67. TUNEL staining of sections was performed using the diaminobenzidine (DAB) (streptavidin and horseradish peroxidase (SA-HRP)) TUNEL Cell Apoptosis Detection Kit (G1507, Servicebio, China) according to the intrustion. Anti-Ki67 mouse monoclonal antibody (mAb; GB121141,Servicebio, China) and immunohistochemistry kits (G1216, Servicebio, China) were used for Ki67 staining of sections. Five randomly view (*n* = 4) for each staining were analyzed semiquantitatively by the ImageJ software.

### 2.4. Real-Time Polymerase Chain Reaction (RT-qPCR)

Total RNA was extracted from lung tissues using TRIzol reagent (423707, Thermo Fisher, USA) and followed by cDNA synthesis using a reverse transcription kit (R333-01, Vazyze, China). The gene expression level of inflammation-related factors and fibrosis-related factors were examined according to the intrustion of the TOROGreen HRM qPCR Master Mix (QET-100, toroivd, China), three replicate wells per sample. Primer sequences are shown in Table 1. Glyceraldehyde-3-phosphate dehydrogenase (GAPDH) was used as an internal reference (*n* = 4).

### 2.5. Western Blotting

The proteins from lung tissue were extracted with radioimmunoprecipitation assay buffer (RIPA) lysate (E122-01, GenStar, China). The proteins were separated by sodium dodecyl sulfate–polyacrylamide gel electrophoresis (SDS-PAGE), and transferred onto a nitrocellulose filter membrane and then blocked with 5% nonfat milk (D8340, Solarbio, China) for 1.5 h. The membranes were incubated overnight at 4°C with primary antibody against Collagen I (1:1000, 14695-1-AP), *α*-smooth muscle actin (*α*-SMA; 1:5000, 14395-1-AP), E-Cadherin (1:10,000, 20874-1-AP), *β*-Tubulin (1:5000, 10094-1-AP). The membranes were then incubated with HRP-conjugated Affinipure Goat Anti-Rabbit immunoglobulin G (IgG; H + L; 1:5000, SA00001-2) or HRP-conjugated Affinipure Goat Anti-Mouse IgG (H + L; 1:5000, SA00001-1) for 1.5 h. All antibodies were purchased from Proteintech (USA). Proteins were visualized in a fully automated luminescence system (Tanon Science and Technology, Tanon 4600SF). At least three repetitions of each effect were performed.

### 2.6. Malondialdehyde (MDA) Concentration Detection

Mouse lung tissue was lysed using RIPA lysate (E122-01, GenStar, China), followed by quantification using the bicinchoninic acid assay (BCA) method. The MDA levels were measured by using Lipid Peroxidation MDA assay kit (S0131S, Beyotime, China) according to the protocol. Three replicate wells were set up for each sample (*n* = 4).

### 2.7. Determination of 8-OHG Using Enzyme-Linked Immunosorbent Assay (ELISA)

To detect 8-OHG, we performed the ELISA method described by Chiou et al. [20] and obtained the 8-OHG-bovine serum albumin (BSA) conjugate using 8-OHG (0496623-22, Cayman Chemical Company, USA) and BSA (SO4S-4 S-S, Amresco, USA). The 8-OHG-BSA conjugates was diluted to 100 ug/mL and then added to the 96-well plates at 100 µL per well and stored overnight at 4°C. Five percent BSA was added and incubated for 1 h at 37°C. One microgram of total RNA extracted from lung tissue (*n* = 4) and 1 μL of 8-OHG antibody (1:500, 10920, Santa Cruz Biotechnology, USA) was added to plate, added RNase-free Water to 100 μL, and incubated at 37°C for 2 h. Then, 100 μL of secondary antibody (1:5000, SA00001-1, Proteintech, USA) was added to each well and incubated at 37°C for 1 h. Add 100 μL tetramethylbenzidine (TMB; A2015024, Aladdin, China) solution, incubate at 37°C for 30 min, and then terminate the reaction. Finally, the absorbance was read at 450 nm using enzyme-labeled instrument.

### 2.8. Reverse Transcription Blocking Combining With Double Primer PCR

Based on the work of Gong, Tao, and Li [21], our laboratory optimized and established the reverse transcription blockade combined double primer amplification method [22]. After optimizing the experimental conditions, reverse transcription was performed using 0.1 μL ImProm-II Reverse Transcriptase (A3803, Promega, USA), which is 10% of the recommended dosage of enzyme, according to our experience. Experiment was performed using the TB Green Master Premix Ex Taq II (Tli RNaseH Plus; RR820A, TaKaRa, Japan) according to the instructions. Two pairs of primers near the either 5′ or 3′ ends were used for each gene, and three replicate wells were set up for each sample (*n* = 4). Primer sequences are shown in Table 1. The degree of RNA oxidative damage was analyzed using the *R* value, which was calculated as follows: *∆Ct* = *Ct* (3′ end) − *Ct* (5′ end), *Rx* (experimental group *R* value) = 2^−*∆Ctx*^, *Ro* (control group *R* value) = 2^−*∆Cto*^, and *R* = *Rx*/*Ro*.

### 2.9. Statistical Analysis

The experimental data were analyzed using GraphPad Prism 9 (GraphPad Software, La Jolla, CA, USA), and the data are expressed as the mean ± standard error of the mean (SEM). One-way analysis of variance (ANOVA) was used to compare the means among three or more experimental groups. *P* < 0.05 was defined as statistically significant.

## 3. Results

### 3.1. MSCs Treatment for RILI

**Figure 1:**
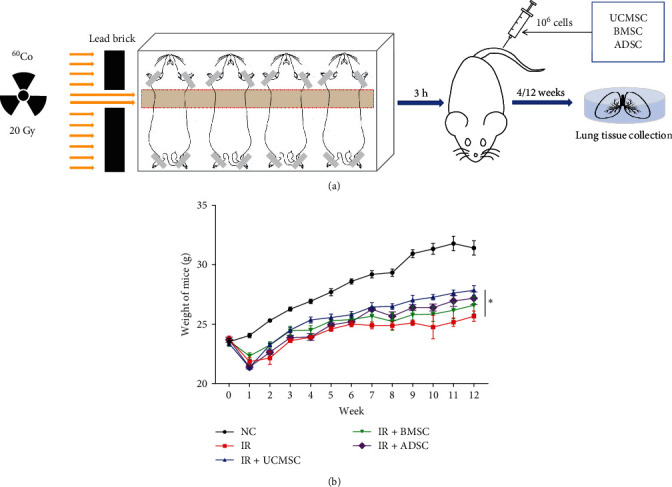
RILI model was developed and treated with UCMSC, BMSC, and ADSC. (a) Procedures for modeling and treating RILI. (b) Changes of body weight in MSC-treated mice within 12 weeks after radiation. ⁣^*∗*^*P* < 0.05. ADSC, adipose-derived stem cell; BMSC, bone marrow mesenchymal stem cell; IR, irradiation group; MSCs, mesenchymal stem cells; NC, normal group; RILI, radiation-induced lung injury; UCMSCs, umbilical cord mesenchymal stem cells.

To investigate the effect of MSCs treatment of RILI, a mouse model of RILI was established by irradiating their chests with ^60^Co while covering the rest of their bodies with lead bricks at a dose of 20 Gy. Three types of MSCs were injected into the tail vein within 3 h, and samples were collected at 4 and 12 weeks after radiation (Figure 1a). After 12 weeks, the irradiation group exhibited a significant decrease in body weight compared to the control group. However, the MSCs-treated group showed a rebound in body weight compared with the irradiation group, with the UCMSCs-treated group demonstrating the most obvious rebound (Figure 1b).

### 3.2. Remediation of Radiation-Induced Pathological Lesions of Lung Tissue by MSCs

**Figure 2:**
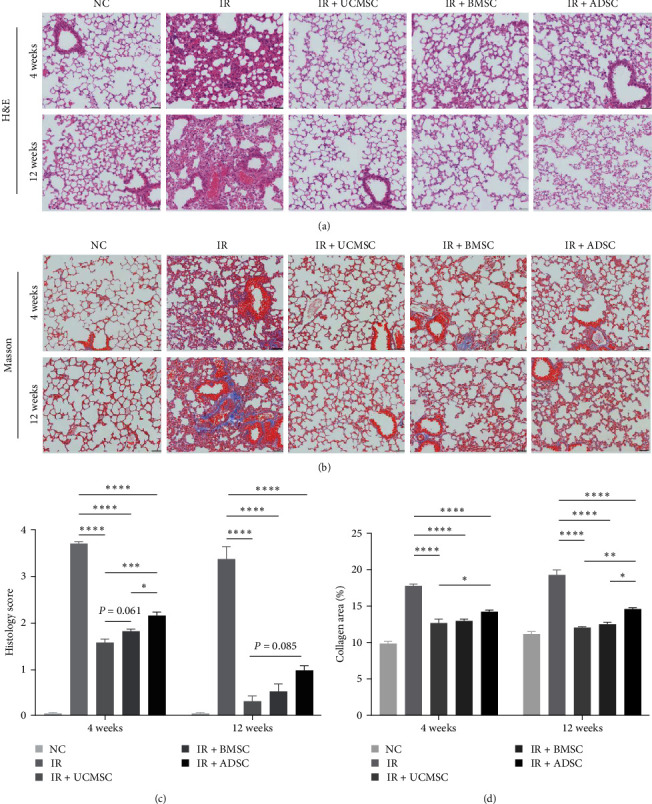
MSCs protects mice from RILI. (a) H&E staining of lungs from mice exposed to irradiation was performed to evaluate the therapeutic effects of UCMSC, BMSC, and ADSC therapies. Representative view of each group is shown. (b) Masson staining was conducted to assess collagen accumulation in mouse lungs after radiation exposure. Representative view of each group is shown. (c) Histology score based on H&E staining was show. (d) Semiquantitative of collagen deposition. *N* = 4. Scale bar = 50 μm. ⁣^*∗*^*P* < 0.05, ⁣^*∗∗*^*P* < 0.01, ⁣^*∗∗∗*^*P* < 0.001, ⁣^*∗∗∗∗*^*P* < 0.0001. ADSC, adipose-derived stem cell; BMSC, bone marrow mesenchymal stem cell; H&E, hematoxylin and eosin; IR, irradiation group; MSCs, mesenchymal stem cells; NC, normal group; RILI, radiation-induced lung injury; UCMSCs, umbilical cord mesenchymal stem cells.

To further understand the pathological changes in the lung tissues of mice after treatment with MSCs, we collected tissue samples at 4 and 12 weeks after irradiation, respectively. H&E staining revealed obvious inflammatory infiltration, thickening of alveolar wall, and alveolar hemorrhage in irradiated mice lungs. However, these effects were mitigated by all three types of MSCs treatment, with UCMSCs exhibiting the most significant improvement (Figure 2a,c). Masson staining demonstrated collagen accumulation in irradiated mouse lung tissue which was alleviated by all three types of MSCs treatment, UCMSCs showed particularly favorable results in the early and late stages of treatment (Figure 2b,d).

### 3.3. Effect of MSCs on Radiation-Induced Cellular Damage in Lung Tissue on Early Stage

**Figure 3:**
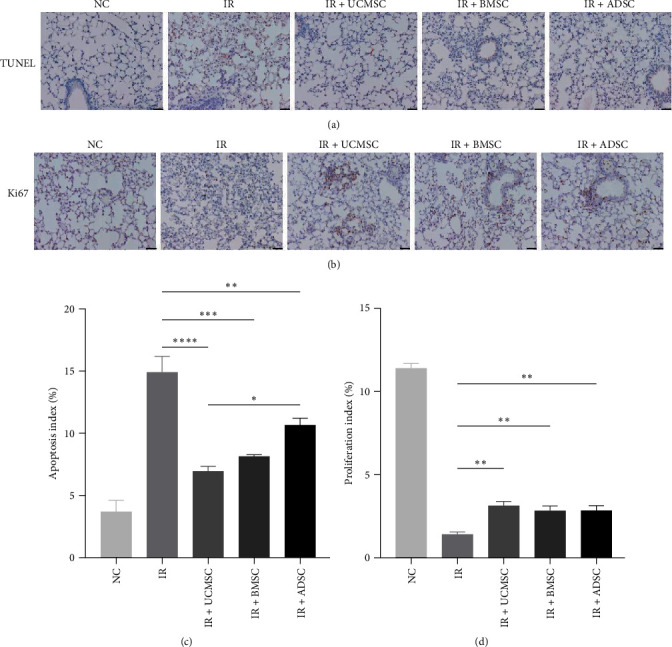
MSCs decreased apoptosis and promoted regeneration in mouse lung tissue 4 weeks after irradiation. (a) TUNEL staining was performed to detect apoptosis in the lung tissue of mice. The picture shows the representative view of each group. (b) Ki67 staining was used to assess the proliferation of mouse lung tissue. The picture shows the representative vision of each group. (c, d) Semiquantitative analysis of TUNEL and Ki67 staining. *N* = 4. Scale bar = 50 μm. ⁣^*∗*^*P* < 0.05, ⁣^*∗∗*^*P* < 0.01, ⁣^*∗∗∗*^*P* < 0.001, ⁣^*∗∗∗∗*^*P* < 0.0001. ADSC, adipose-derived stem cell; BMSC, bone marrow mesenchymal stem cell; IR, irradiation group; MSCs, mesenchymal stem cells; NC, normal group; TUNEL, terminal deoxynucleotidyl transferase-mediated dUTP nick end labeling; UCMSCs, umbilical cord mesenchymal stem cells.

Radiation causes apoptosis of lung tissue cells and exacerbates lung injury [10]. After 4 weeks of irradiation, TUNEL immunohistochemical staining revealed a marked increase in apoptosis among lung tissue cells; however, this radiation-induced increase of apoptosis was reversed by MSCs treatment which represents rescuing damaged lung tissue cells from death, which exhibits better therapeutic efficacy in UCMSCs treatment than ADSCs treatments (Figure 3a,c). Ki67 staining indicated that the proliferation of lung tissue cell was also significantly reduced after irradiation, which impeded the regeneration of lung tissue after injury, but all three MSCs treatments were effective in promoting tissue-promoting regeneration (Figure 3b,d). These results suggest that MSCs may protect mice from radiation-induced cellular damage in lung tissue at an early stage of RILI.

### 3.4. MSCs Attenuate RILI by Modulating Inflammation-Related Factors

**Figure 4:**
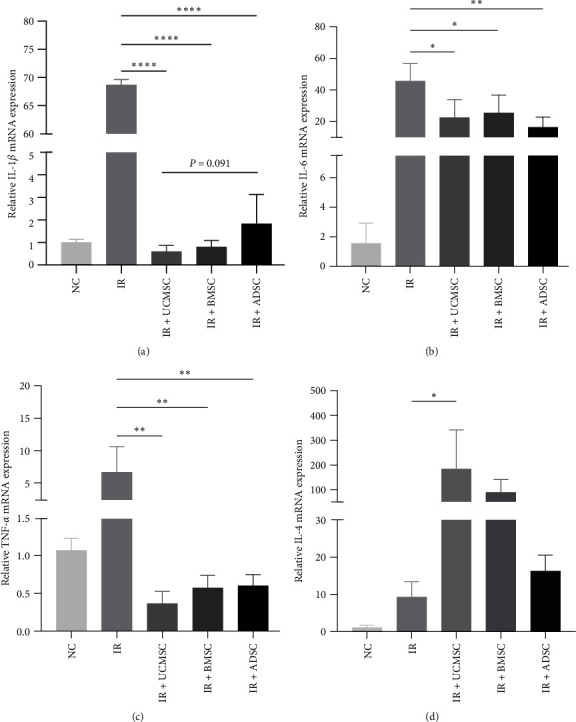
MSCs reduced RILI by decreasing inflammatory factors and increasing anti-inflammatory factors. RT-PCR detected the expression levels of IL-1*β* (a), IL-6 (b), TNF-*α* (c), and IL-4 (d) mRNA in mouse lung tissues 4 weeks after radiation. *N* = 4. ⁣^*∗*^*P* < 0.05, ⁣^*∗∗*^*P* < 0.01, ⁣^*∗∗∗∗*^*P* < 0.0001. ADSC, adipose-derived stem cell; BMSC, bone marrow mesenchymal stem cell; IL-1*β*, interleukin-1 beta; IL-4, interleukin-4; IL-6, interleukin-6; IR, irradiation group; MSCs, mesenchymal stem cells; NC, normal group; RILI, radiation-induced lung injury; TNF-*α*, tumor necrosis factor- alpha; UCMSCs, umbilical cord mesenchymal stem cells.

To further investigate the therapeutic effects and mechanisms of the three types of MSCs treatment, RT-PCR analysis was conducted to measure expression levels of cytokines within the lung tissues. Results demonstrated that the level of inflammatory factors interleukin-1 beta (IL-1*β*), interleukin-6 (IL-6), and tumor necrosis factor-alpha (TNF-*α*) were significantly upregulated in mouse lung tissues 4 weeks after irradiation (Figure 4a−c), there was a significant downregulation in MSCs treatment groups. UCMSCs were able to significantly upregulate the anti-inflammatory factor interleukin-4 (IL-4; Figure 4d) and showed a better ability to alleviate inflammation.

### 3.5. MSCs Attenuate RILI by Regulating the Fibrotic Process

**Figure 5:**
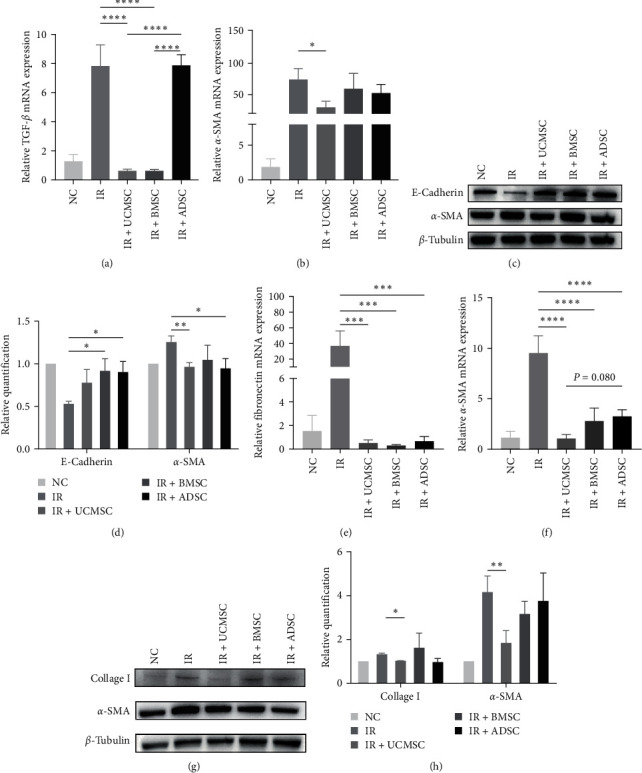
MSCs reduced RILI by modulating fibrosis-related factors. RT-PCR detected the expression levels of TGF-*β* (a) and *α*-SMA (b) mRNA in mouse lung tissues 4 weeks after radiation. *N* = 4. (c, d) Western blot and relative qualification of E-Cadherin and *α*-SMA protein levels in mouse lung tissues 4 weeks after irradiation. *N* ≥ 3. RT-PCR detected the expression levels of fibronectin (e) and *α*-SMA (f) mRNA in mouse lung tissues 12 weeks after radiation. *N* = 4. (g, h) Western blot and relative qualification of collagen I and *α*-SMA protein levels in mouse lung tissues 12 weeks after irradiation. *N* ≥ 3. ⁣^*∗*^*P* < 0.05, ⁣^*∗∗*^*P* < 0.01, ⁣^*∗∗∗*^*P* < 0.001, ⁣^*∗∗∗∗*^*P* < 0.0001. ADSC, adipose-derived stem cell; BMSC, bone marrow mesenchymal stem cell; IR, irradiation group; MSCs, mesenchymal stem cells; NC, normal group; RILI, radiation-induced lung injury; TGF-*β*, transforming growth factor-beta; UCMSCs, umbilical cord mesenchymal stem cells.

Radiation causes fibrosis in lung tissue, and in order to understand the effect of MSCs on lung fibrosis, we examined fibrosis-related factors in lung tissue. RT-PCR analysis showed that the expression of transforming growth factor-beta (TGF-*β*) and *α*-SMA, which are thought to cause fibrosis [23], was upregulated in lung tissue after 4 weeks of irradiation, and UCMSC significantly reduced this change; compared to ADSC, UCMSC, and BMSC treatments played a more significant role in reducing TGF-*β* (Figure 5a,b). Western blot analysis showed that after 4 weeks of irradiation, the epithelial marker E-Cadherin was downregulated and the mesenchymal marker *α*-SMA was upregulated, promoting epithelial–mesenchymal transition, which has been shown to promote fibrosis [24]; however, MSCs treatments prevented this process (Figure 5c,d). RT-PCR analysis showed that the expression of fibronectin and *α*-SMA, which are thought to cause fibrosis [25, 26], were upregulated after 12 weeks of irradiation, further suggesting that radiation promotes fibrosis through epithelial–mesenchymal transition and that MSC therapy can effectively modulate this process (Figure 5e,f). Furthermore, Western blot detection of *α*-SMA and collagen I at 12 weeks after irradiation demonstrated their upregulation along with collagen accumulation leading to advanced lung fibrosis. However, MSCs treatment mitigated these effects to attenuate radiation-induced lung fibrosis, with UCMSCs treatment playing a significant role (Figure 5g,h). Taken together, MSCs may attenuate lung injury at all stages of RILI by regulating the expression of fibrosis-related factors.

### 3.6. MSCs Decrease the Level of Oxidized RNA in Lung Tissue With RILI

**Figure 6:**
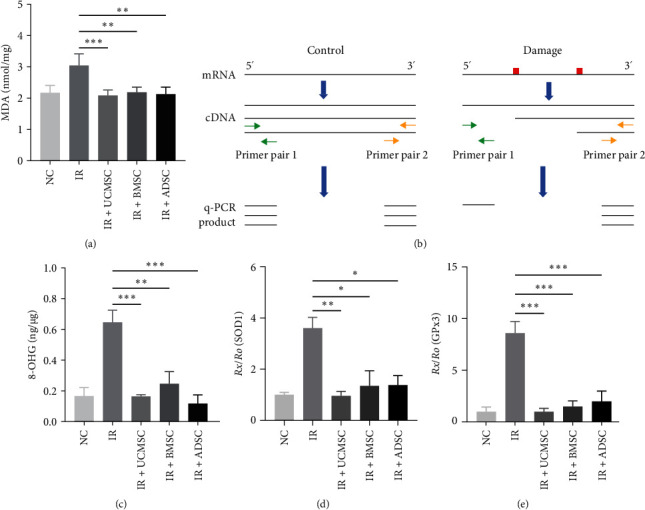
MSCs reduced RILI by decreasing RNA oxidative damage. (a) The MDA level in lung tissues was determined. (b) The 8-OHG level in total RNA extracted from lung tissues was detected by ELISA. (c) Flowchart of reverse transcription blocking combining with double primer PCR experiment. (d) Reverse transcription blocking combining with double primer PCR was utilized to detect SOD1 mRNA damage. (e) Reverse transcription blocking combining with double primer PCR was utilized to detect GPx3 mRNA damage. *N* = 4. ⁣^*∗*^*P* < 0.05, ⁣^*∗∗*^*P* < 0.01, ⁣^*∗∗∗*^*P* < 0.001. 8-OHG, 8-hydroyguanosine; ADSC, adipose-derived stem cell; BMSC, bone marrow mesenchymal stem cell; ELISA, enzyme-linked immunosorbent assay; GPx3, glutathione peroxidase 3; IR, irradiation group; MDA, malondialdehyde; MSCs, mesenchymal stem cells; NC, normal group; RILI, radiation-induced lung injury; SOD1, superoxide dismutase 1; UCMSCs, umbilical cord mesenchymal stem cells.

Following radiation exposure, the lung tissue generates a significant amount of ROS [27], which leads to lipid damage and subsequent production of MDA [28]. We found that all three MSC therapies were effective in reducing radiation-induced increases in MDA in lung tissue (Figure 6a). We have previously shown that ROS can cause RNA damage in vitro (unpublished data), and literature reports also suggest that ROS may be the primary cause of RNA oxidation [29]. Therefore, we hypothesized that RNA damage may also be existed in RILI; however, little attention has been given to radiation-induced RNA damage. Among various forms of RNA damage, 8-OHG is the most common type [30]. We found that the level of 8-OHG in total RNA of irradiated lung tissues was significantly upregulated by ELISA experiments, while all three MSC treatments resulted in its downregulation (Figure 6b). To assess mRNA damage in specific genes, we employed reverse transcription blocking combined with double-primer PCR assay. This method relies on the principle that oxidative damage to RNA hinders proper reverse transcription and results in different product quantities at the 5′ end compared to the 3′ end (Figure 6c). We evaluated mRNA damage in glutathione peroxidase 3 (GPx3) and superoxide dismutase 1 (SOD1) genes, both of which are critical for ROS scavenging [31, 32]. The results showed that the mRNA damage of GPx3 and SOD1 increased eightfold and threefold, respectively, after irradiation, but was significantly reduced by treatment with MSCs to levels comparable to the control (Figure 6d,e).

To summarize, the findings indicate that MDA, 8-OHG, SOD1, and GPx3 mRNA damage exhibit significant upregulation following irradiation, but can be effectively restored through MSC treatment. These results provide compelling evidence for the potential of MSCs therapy in the complete elimination of RNA damage induced by ionizing radiation in RILI.

## 4. Discussion

MSCs is one of the most promising the treatment of RILI [33]. In this study, three types of MSCs can alleviate the pathological damage of lung tissue induced by ^60^Co radiation, such as inflammatory infiltration, alveolar wall thickening, and alveolar hemorrhage. They affect the radiation-induced inflammatory response by reducing inflammatory factors and upregulating anti-inflammatory factors and the fibrotic response by regulating fibrosis-related factors. In addition, our preliminary explorations revealed that radiation could cause oxidative damage to RNA in lung tissue, and the level of 8-OHG was significantly upregulated after radiation. We found that the mRNA of SOD1 and GPx3, the key enzymes for scavenging ROS, were damaged by oxidation, which may be one of the reasons for the downregulation of SOD1 protein after radiation damage [18], and MSC therapy significantly attenuated RNA damage.

In this study, the therapeutic effects of human UCMSCs, BMSCs, and ADSCs on RILI were simultaneously compared for the first time, and the therapeutic effects of UCMSCs were found to be relatively better. Previous studies have shown that UCMSCs are more effective than blood-derived MSCs on treating lipopolysaccharide-induced acute lung injury in mice [34]; and BMSCs and ADSCs were more effective than lung MSCs in treating lipopolysaccharide-induced acute respiratory distress syndrome in rats [35], UCMSCs expressed more secretory factors and could better promote vascular regeneration than BMSCs [36]. We found that all three types of MSCs could better treat radiation-induced lung tissue pathological injury, and the therapeutic effect of UCMSC was the most significant. The administration of UCMSC treatment demonstrated a significant reduction in radiation-induced apoptosis in lung tissue. Additionally, it demonstrated significant efficacy in upregulating the anti-inflammatory factor IL-4 compared to BMSC and ADSC treatment. Studies have shown that the development of epithelial–mesenchymal transition contributes to lung fibrosis [37]. However, UCMSC treatment effectively reduces fibrosis through its therapeutic effects by reducing the expression of the *α*-SMA and collagen I protein. Moreover, UCMSCs possess the advantage of being obtained from a wide range of sources, making them more suitable for implementation in the treatment of RILI.

Radiation affects DNA structure directly by inducing DNA breaks and indirectly by generating ROS, which in turn damage DNA molecules [38]. ROS originating from cells impair DNA through diverse mechanisms, including base damage, base release, depolymerization, cross-linking, and strand breaks [39]. Conversely, limited attention has been given to radiation-induced RNA damage. RNA, being single-stranded and predominantly localized in the cytoplasm, is more susceptible to ROS exposure. Furthermore, in addition, RNA that is less bound to proteins is more susceptible to oxidative damage [40]. Previous research has demonstrated that greater RNA than DNA oxidation was induced in rat liver after doxorubicin administration [41, 42] and that RNA damage surpasses DNA damage in lung epithelial cell injury induced by hydrogen peroxide [43]. However, the extent of RNA oxidation in RILI remains unexplored. Numerous studies have established the significance of RNA oxidation in various diseases, including atherosclerosis [44], myopathy [45], Alzheimer's disease [46, 47], and Parkinson's disease [48, 49]. In this study, the level of oxidative damage to cellular RNA in the lung tissue of mice with RILI was investigated for the first time, the findings revealed a significant upregulation of 8-OHG, indicating heightened levels of RNA oxidation. In our study, it was observed that the damaged mRNA levels of SOD1 and GPx3, which are crucial enzymes involved in scavenging ROS, were increased in the lung tissue of irradiated mice. This suggests that radiation may directly impair RNAs, while concurrently triggering the production of substantial quantities of ROS-damaging mRNAs, such as those encoding SOD1 and GPx3. Consequently, reduced levels of these key ROS scavenging enzymes lead to the accumulation of ROS in tissues. The administration of MSCs treatment has been found to mitigate this phenomenon. Previous research has demonstrated that MSCs exosomes possess the ability to decrease mitochondrial ROS levels induced by lung injury in acute respiratory distress syndrome [50] and chronic obstructive pulmonary disease [51]. The effectiveness of MSC therapy in reducing RNA oxidation may be attributed to their ability to reduce ROS. This provides new evidence for the use of MSC in treating RILI and suggests their potential in treating RNA oxidation-related diseases.

## 5. Conclusions

Therapeutic effects of human UCMSCs, BMSCs, and ADSCs on RILI in mice were simultaneously compared, and based on the therapeutic effects and MSCs origin, we concluded that UCMSC was more advantageous. In our preliminary examination, we observed that radiation resulted in a significant upregulation of 8-OHG content and the presence of damaged mRNAs of SOD1 and GPx3 in lung tissue of mice. Three types of MSCs treatments demonstrated significant reduction of oxidative damage in RILI. However, further comprehensive investigation is required to thoroughly understand the role and mechanism of RNA oxidation damage in the treatment of RILI using MSCs.

## Figures and Tables

**Table 1:** Primers used for PCR.

Primer name	Forward primer sequence (5′ to 3′)	Reverse primer sequence (5′ to 3′)
TNF-*α*	CGCTGAGGTCAATCTGC	GGCTGGGTAGAGAATGGA
IL-1*β*	CCAAGCTTCCTTGTGCAAGTA	AAGCCCAAAGTCCATCAGTGG
IL-6	ACAGAAGGAGTGGCTAAGGA	AGGCATAACGCACTAGGTTT
GAPDH	CATCACTGCCACCCAGAAGACTG	ATGCCAGTGAGCTTCCCGTTCAG
TGF-*β*	TGATACGCCTGAGTGGCTGTCT	CACAAGAGCAGTGAGCGCTGAA
Fibronectin	CCCTATCTCCTGATACCGTGTGTCC	TGCCGCAACTGTGATTCGG
*α*-SMA	TGCTGACAGAGGCACCACTGAA	CAGTTGTACGTCCAGAGGCATAG
IL-4	ATCATCGGCATTTTGAACGAGGTC	ACCTTGGAAGCCCTACAGACGA
SOD1-5′	GATGAAAGCGGTGTGCGTG	GTTCACCGCTTGCCTTCTG
SOD1-3′	CGTACAATGGTGGTCCATGA	GCTCCCAGCATTTCCAGTCT
GPX3-5′	CATCCTGCCTTCTGTCCCTG	CGCCATGGCAGTCTGTCTTA
GPX3-3′	TCTACACTTTCCTGAAGAACTCCTG	GACGTTGCTGACTGTGGTCC

Abbreviations: *α*-SMA, *α*-smooth muscle actin; GAPDH, glyceraldehyde-3-phosphate dehydrogenase; GPx3, glutathione peroxidase 3; IL-1*β*, interleukin-1 beta; IL-4, interleukin-4; IL-6, interleukin-6; SOD1, superoxide dismutase 1; TGF-*β*, transforming growth factor-beta; TNF-*α*, tumor necrosis factor-alpha.

## Data Availability

The data used to support the findings of this study are included within the article.
